# Neuroimmune cardiovascular interfaces in atherosclerosis

**DOI:** 10.3389/fcell.2023.1117368

**Published:** 2023-01-30

**Authors:** Sarajo K. Mohanta, Changjun Yin, Christian Weber, Andreas J. R. Habenicht

**Affiliations:** ^1^ Institute for Cardiovascular Prevention, Ludwig-Maximilians-Universität München (LMU), Munich, Germany; ^2^ German Center for Cardiovascular Research (DZHK), Partner Site Munich Heart Alliance, Munich, Germany; ^3^ Institute of Precision Medicine, The First Affiliated Hospital of Sun Yat-sen University, Guangzhou, Guangdong, China; ^4^ Munich Cluster for Systems Neurology (SyNergy), Munich, Germany

**Keywords:** neuroimmune cardiovascular interface, neuroimmunology, neurovascular link, adventitia, atherosclerosis, artery tertiary lymphoid organs

## Abstract

Two pairs of biological systems acting over long distances have recently been defined as major participants in the regulation of physiological and pathological tissue reactions: i) the nervous and vascular systems form various blood-brain barriers and control axon growth and angiogenesis; and ii) the nervous and immune systems emerge as key players to direct immune responses and maintain blood vessel integrity. The two pairs have been explored by investigators in relatively independent research areas giving rise to the concepts of the rapidly expanding topics of the neurovascular link and neuroimmunology, respectively. Our recent studies on atherosclerosis led us to consider a more inclusive approach by conceptualizing and combining principles of the neurovascular link and neuroimmunology: we propose that the nervous system, the immune system and the cardiovascular system undergo complex crosstalks in tripartite rather than bipartite interactions to form neuroimmune cardiovascular interfaces (NICIs).

## The neurovascular link

Reviewing interactions between the nervous system and the cardiovascular system, Carmeliet and Tessier-Lavigne recently pointed out the significance of observations made by the Belgian anatomist Andreas Vesalius in 1543 for our current views of interactions between the nervous and cardiovascular systems (Carmeliet and Tessier-Lavigne, 2005). Indeed, the studies by Vesalius turn out to be highly relevant for our current understanding of the common mechanisms underlying nerve and blood vessel development. He was the first to point to the striking macroanatomical proximity of the peripheral nervous system and the vascular system. Moreover, recent research identified a multitude of cardiovascular-nervous system interactions leading to discoveries of countless functionally relevant mediators released from either tissue and acting on the other. Likewise, basic research into the evolution of neurogenesis, immune system development and angiogenesis in small animals (Zacchigna et al., 2008) from nematode worms (*Caenorhabditis elegans*) to fruit flies (*Drosophila melanogaster*) to zebra fish (*Danio rerio*) have expanded current notions regarding the functionality of the relationships of three systemically acting tissues: the nervous system, the immune system and the cardiovascular system. Whereas *C. elegans* have developed a well-organized nervous system consisting of 302 stereotyped neurons (of a total of 959 somatic cells), they lack a cardiovascular system. However, they have acquired an emerging primitive innate immune system consisting of sessile muscle cells that are capable of phagocytosis of danger-signaling macromolecules including infectious organisms. Of note, this nematode innate immune system is regulated by neuronal inputs (Liu and Sun, 2021). Next in the phylogenetic tree of invertebrate multicellular organisms considered here, are fruit flies which own a more advanced and well-developed nervous system (∼100,000 neurons) and an emerging innate non-sessile circulating immune system in their haemolymph which can be regarded as a primitive circulatory system. Finally, zebrafish feature all three systems at advanced stages of development including a well-developed regularly beating heart (Jopling et al., 2010). In particular, the zebrafish turned out to be an important model for human heart development and a series of heart diseases including aorta calcification (Singh et al., 2019). Furthermore, the zebrafish allows to examine heart development with a speed and information not achievable even in mouse models. Indeed, zebrafish allow to study neural-cardiovascular connections to define the role of every single nerve from the heart and the cardiovascular system (Vedder et al., 2020). Fishman et al. pioneered the zebrafish as a model, generated multiple genetically altered fish and applied them to human disease using powerful genetic screens (Lee et al., 1994; Weinstein et al., 1995; Stainier et al., 1996; Fouquet et al., 1997; Childs et al., 2002). When taken together, these data revealed that the nervous system appeared first during evolution of multiple species followed by the immune system and the cardiovascular system (Tam and Watts, 2010; Brunet et al., 2014). Recent progress in understanding the neurovascular link in the central nervous system and in the peripheral nervous system, respectively, expands our perceptions of the functional implications in physiology (Walchli et al., 2015). Moreover, the relevance of multi-tissue interactions of the three tissues to understand disease pathogeneses as varied as atherosclerosis, Alzheimer′s Disease (Yin et al., 2019) and Diabetes Mellitus (Malheiro et al., 2021) become increasingly apparent at a rapid pace.

## Neuroimmunology

Parallel to the field of the neurovascular link, a pair of two further systemically interacting biological systems, i.e., the nervous and immune systems, burgeoned during the last decades giving rise to the ever growing - and indeed exploding - field of neuroimmunology. Key growth and survival signals including mediators of neuron neogenesis, axon growth, synaptogenesis, target innervation, nerve growth factor-dependent neuron survival among others and multiple mediators of blood vessel morphogenesis derived from both the nervous and the immune systems have been identified (Glebova and Ginty, 2005). Many, if not all, of these mediators also turn out to participate in diseases as varied as cancer, autoimmune diseases, multiple chronic inflammatory diseases, degenerative and inflammatory brain diseases, bacterial infectious diseases and peripheral nervous system-associated diseases (Andersson and Tracey, 2012; Chiu et al., 2012; Olofsson et al., 2012; Steinman, 2012; Tracey, 2012; Magnon et al., 2013; Han et al., 2015; Hanoun et al., 2015; McMahon et al., 2015; Talbot et al., 2016; Chu et al., 2020; Cserep et al., 2020; Udit et al., 2022). As a result of rather recent studies, neuroimmunology has now reached the realm of neuronal control by distinct brain nuclei and indeed neuron subtypes to regulate peripheral immune responses during physiology and pathophysiology (Tracey, 2012; Magnon et al., 2013). In addition to identifying afferent sensory nervous system-brain axes, regulatory efferent brain-peripheral organ projections are being elucidated (Weinstein et al., 1995; Andersson and Tracey, 2012; Steinman, 2012; Walchli et al., 2015). A striking recent example of this emerging area of neuroimmunology has been the regulation of humoral immune responses in the spleen by distinct cortico-releasing hormone-expressing neurons in the central amygdala and the parabrachial nucleus that instruct the splenic nerve which in turn regulates plasma cell abundance (i.e., B cell immunity) (Zhang et al., 2020). These studies indicate that we are on the cusp of better understanding the dominant roles of the brain in regulating fundamental biological systems in the periphery and the brain itself (already apparent in nematodes as outlined above) including phenomena as diverse as thermoregulation (Morrison and Nakamura, 2019), emotions and empathy (Gehrlach et al., 2019; Zych and Gogolla, 2021) and gut inflammation (Koren et al., 2021; Brea and Veiga-Fernandes, 2022). It becomes apparent that these advances include previously unknown phenomena whose molecular underpinnings had escaped any level of comprehension in the past: We would emphasize the work of Anderson and Adolphs who recently pointed to the possibility that even insects express emotions that they termed *emotion primitives* by citing Darwin′s comment “*Even insects express anger, terror, jealousy and love, by their stridulation*” (Anderson and Adolphs, 2014). Indeed, a key brain area termed the *insular cortex* has recently been identified as a maintenance and integration structure for fear and empathy in mice (Gogolla, 2017; Klein et al., 2021). When taken together, neuroimmunology is now identifying new brain-controlled peripheral biological systems of major significance in physiology and disease. The vascular immune crosstalk has been extensively described elsewhere (Yin et al., 2017; Klein et al., 2021; Brea and Veiga-Fernandes, 2022) and is not the focus of this mini-review.

## Long distance talk of three companions in atherosclerosis

Early studies of our group observed that the majority of immune cells in late stage atherosclerosis (Libby et al., 2011) accumulated in the lamina adventitia, i.e. the connective tissue coat of arteries, rather than in intima plaques. Some of the immune cell aggregates had a large B cell component particularly in areas of heavily diseased artery segments. To our initial surprise, these aggregates resembled *tertiary lymphoid organs* that had previously reported in distinct types of cancer, autoimmune diseases and chronic unresolvable inflammatory diseases (Brea and Veiga-Fernandes, 2022). Following a series of imaging and functional experiments, we termed these atherosclerosis-associated aggregates *artery tertiary lymphoid organs* (ATLOs) (Grabner et al., 2009; Yin et al., 2017). We phenotyped ATLOs and found that they harbor multiple B cell subtypes including germinal center B cells, B1 cells in activated B cell follicles, both short-lived and long-lived plasma cells in separate niches and also separate T cell areas in addition to innate immune cells (Srikakulapu et al., 2016). Subsequent *in vitro* studies revealed that stimulation of the lymphotoxin β receptor on arterial smooth muscle cells changed their phenotype to resemble cells that had been termed *lymphoid organizer cells* as they expressed the lymphorganogenic chemokines CXCL13 and CCL19 (Mohanta et al., 2022; Hu et al., 2019). These *in vitro* data together with the observation that arterial media smooth muscle cells adjacent to atherosclerotic plaques *in vivo* showed strong CXCL13 expression led us to generate transgenic mice with a selective deletion of the lymphotoxin β receptor in the smooth muscle cells. In aged hyperlipidemic smooth muscle cell-specific lymphotoxin β receptor-deletion mice, the extent of atherosclerosis burden was higher than in their hyperlipidemic counterparts. These data indicated that ATLOs may affect atherosclerosis progression (Hu et al., 2015). Meanwhile, Akhavanpoor et al. (2018) observed ATLO formation in human atherosclerotic coronary arteries: They reported that all patients with myocardial infarction showed ATLOs stage-III in their coronary artery adventitia that we had previously defined as large T/B cell aggregates containing follicular dendritic cells in germinal centers. More recently, we isolated ATLO and plaque T cells, analyzed them using a pairing approach of single cell RNA sequencing (scRNA-seq) with single cell T cell receptor sequencing and observed clonal expansion of CD4, T regulatory and CD8 T cells. These data showed the power of scRNA-seq approaches to understand the mechanisms of atherosclerosis (Wang et al., 2022; Zhang et al., 2022).

In parallel studies, we searched for molecular cues of a poorly understood form of dementia, i.e., vascular dementia, that had previously be linked to Alzheimer′s disease: Distinct brain areas including the choroid plexus and areas heavily burdened by Alzheimer plaques in mouse and human brains were examined in parallel with murine and human atherosclerotic plaques. Interestingly, the classical complement component C1q was found to form high-affinity complexes with Apolipoprotein E and inhibition of the C1q-mediated inflammatory pathway using siRNA treatment in mice reduced both atherosclerosis and Alzheimer plaque (intermediate) burdens (Yin et al., 2019). Using laser capture microdissection-based whole genome-wide transcript profiling, we observed a major and dominant interferon signature in the choroid plexus that was dependent on the transgenic expression of the Apolipoprotein E4 isoform in transgenic knock-in mice maintained on a Western type diet indicating that the genetics of key regulatory genes in atherosclerosis progression deserve attention. These findings directly associated Apolipoprotein E and its isoforms with major brain diseases in which neuroimmune responses play major roles (Yin et al., 2019). As the adventitia forms the major conduit for the nervous system to reach distant targets (as Vesalius noted, see above) in hyperlipidemic mice during aging (Moos et al., 2005; Mohanta et al., 2014), we hypothesized that atherosclerosis-specific adventitia segments may interact with the nervous system.

**FIGURE 1:**
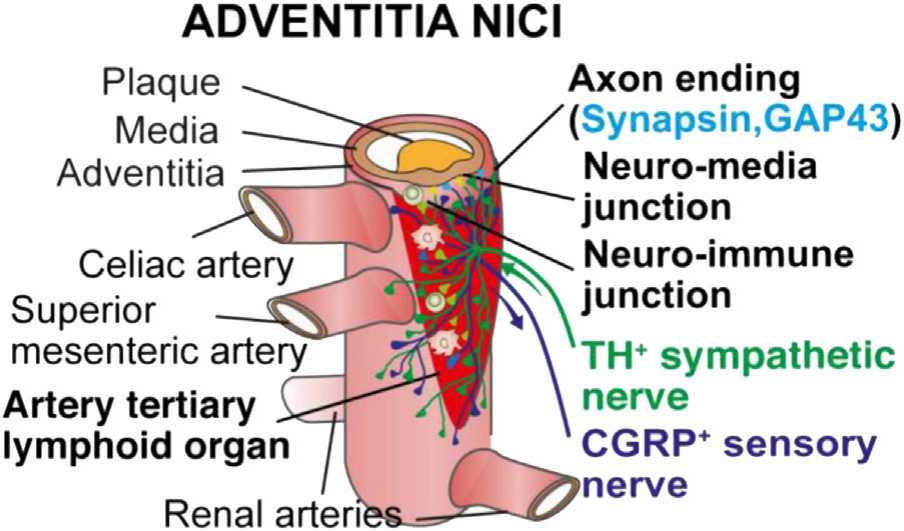
Choreography of an adventitia NICI in advanced murine atherosclerosis. Recent studies into a possible innervation of the adventitia by the axons of the peripheral nervous system revealed that both the sensory and the sympathetic nervous systems undergo marked restructuring in artery segments afflicted with atherosclerosis in murine models of atherosclerosis and human adventitia segments adjacent to plaques in multiple territories of the arterial tree [adopted from (Mohanta et al., 2022)].

All these studies raised the possibility that ATLOs may be a model to study how the atherosclerotic arterial wall may directly crosstalk with the peripheral nervous system. Using a multitude of imaging methods including tissue clearing, we recently observed that the components of the peripheral nervous system in the artery adventitia adjacent to atherosclerotic plaques underwent marked restructuring (Mohanta et al., 2022; Sun et al., 2022). Atherosclerosis-triggered restructuring included axon outgrowth, formation of junction-like synaptic connections between immune cells and axons and formation of growth cones among others (Figure 1). Moreover, surgical removal of the sympathetic celiac ganglia in the abdominal portion of the aorta in the aged mice attenuated the burden of atherosclerosis in this segment (Mohanta et al., 2022). These morphological and functional studies led us to propose that the diseased arterial wall directly crosstalks to both the immune and the nervous system in tripartite rather than bidirectional interactions. In addition to the adventitia NICI depicted below, other changes were noticed in murine and human atherosclerosis including inflammatory infiltrates around peripheral nervous system ganglia and nerves. We therefore suggest that the tripartite tissue interaction supports the existence of a until now underappreciated disease paradigm (Mohanta et al., 2022).

## Future perspectives

Future work should be directed towards a more detailed understanding of the morphology and function of the adventitia and other peripheral nervous system NICIs. These studies would characterize the axon tips in the adventitia of diseased artery segments using quantitative electron microscopy and morphologically delineate the neuroimmune junctions. Moreover, a more complete functional characterization of the sensory nervous system including the pain receptors/nociceptors on atherosclerosis progression may yield new information on the adventitia NICI when scRNA-seq approaches are employed (Wang, 2023). We also propose to interrogate the NICI hypothesis in diseases other than atherosclerosis including cancer, rheumatoid arthritis and autoimmune diseases that are associated with TLOs.

## References

[B1] Akhavanpoor M. Gleissner C. A. Akhavanpoor H. Lasitschka F. Doesch A. O. Katus H. A. (2018). Adventitial tertiary lymphoid organ classification in human atherosclerosis. Cardiovasc Pathol. 32, 8–14. 10.1016/j.carpath.2017.08.002 29078120

[B2] Anderson D. J. Adolphs R. (2014). A framework for studying emotions across species. Cell 157, 187–200. 10.1016/j.cell.2014.03.003 24679535 PMC4098837

[B3] Andersson U. Tracey K. J. (2012). Reflex principles of immunological homeostasis. Annu. Rev. Immunol. 30, 313–335. 10.1146/annurev-immunol-020711-075015 22224768 PMC4533843

[B4] Brea D. Veiga-Fernandes H. (2022). Inflammation in the gut is encoded by neurons in the brain. Nature 602, 217–218. 10.1038/d41586-021-03802-x 35013603

[B5] Brunet I. Gordon E. Han J. Cristofaro B. Broqueres-You D. Liu C. (2014). Netrin-1 controls sympathetic arterial innervation. J. Clin. investigation 124, 3230–3240. 10.1172/JCI75181 PMC4071369 24937433

[B6] Carmeliet P. Tessier-Lavigne M. (2005). Common mechanisms of nerve and blood vessel wiring. Nature 436, 193–200. 10.1038/nature03875 16015319

[B7] Childs S. Chen J. N. Garrity D. M. Fishman M. C. (2002). Patterning of angiogenesis in the zebrafish embryo. Development 129, 973–982. 10.1242/dev.129.4.973 11861480

[B8] Chiu I. M. von Hehn C. A. Woolf C. J. (2012). Neurogenic inflammation and the peripheral nervous system in host defense and immunopathology. Nat. Neurosci. 15, 1063–1067. 10.1038/nn.3144 22837035 PMC3520068

[B9] Chu C. Artis D. Chiu I. M. (2020). Neuro-immune interactions in the tissues. Immunity 52, 464–474. 10.1016/j.immuni.2020.02.017 32187517 PMC10710744

[B10] Cserep C. Posfai B. Lenart N. Fekete R. Laszlo Z. I. Lele Z. (2020). Microglia monitor and protect neuronal function through specialized somatic purinergic junctions. Science 367, 528–537. 10.1126/science.aax6752 31831638

[B11] Fouquet B. Weinstein B. M. Serluca F. C. Fishman M. C. (1997). Vessel patterning in the embryo of the zebrafish: Guidance by notochord. Dev. Biol. 183, 37–48. 10.1006/dbio.1996.8495 9119113

[B12] Gehrlach D. A. Dolensek N. Klein A. S. Roy Chowdhury R. Matthys A. Junghanel M. (2019). Aversive state processing in the posterior insular cortex. Nat. Neurosci. 22, 1424–1437. 10.1038/s41593-019-0469-1 31455886

[B13] Glebova N. O. Ginty D. D. (2005). Growth and survival signals controlling sympathetic nervous system development. Annu. Rev. Neurosci. 28, 191–222. 10.1146/annurev.neuro.28.061604.135659 16022594

[B14] Gogolla N. (2017). The insular cortex. Curr. Biol. CB 27, R580–R586. 10.1016/j.cub.2017.05.010 28633023

[B15] Grabner R. Lotzer K. Dopping S. Hildner M. Radke D. Beer M. (2009). Lymphotoxin beta receptor signaling promotes tertiary lymphoid organogenesis in the aorta adventitia of aged ApoE-/- mice. J. Exp. Med. 206, 233–248. 10.1084/jem.20080752 19139167 PMC2626665

[B16] Han S. Soleiman M. T. Soden M. E. Zweifel L. S. Palmiter R. D. (2015). Elucidating an affective pain circuit that creates a threat memory. Cell 162, 363–374. 10.1016/j.cell.2015.05.057 26186190 PMC4512641

[B17] Hanoun M. Maryanovich M. Arnal-Estape A. Frenette P. S. (2015). Neural regulation of hematopoiesis, inflammation, and cancer. Neuron 86, 360–373. 10.1016/j.neuron.2015.01.026 25905810 PMC4416657

[B18] Hu D. Mohanta S. K. Yin C. Peng L. Ma Z. Srikakulapu P. (2015). Artery tertiary lymphoid organs control aorta immunity and protect against atherosclerosis via vascular smooth muscle cell lymphotoxin beta receptors. Immunity 42, 1100–1115. 10.1016/j.immuni.2015.05.015 26084025 PMC4678289

[B19] Hu D. Yin C. Luo S. Habenicht A. J. R. Mohanta S. K. (2019). Vascular smooth muscle cells contribute to atherosclerosis immunity. Front. Immunol. 10, 1101. 10.3389/fimmu.2019.01101 31164888 PMC6534067

[B20] Jopling C. Sleep E. Raya M. Marti M. Raya A. Izpisua Belmonte J. C. (2010). Zebrafish heart regeneration occurs by cardiomyocyte dedifferentiation and proliferation. Nature 464, 606–609. 10.1038/nature08899 20336145 PMC2846535

[B21] Klein A. S. Dolensek N. Weiand C. Gogolla N. (2021). Fear balance is maintained by bodily feedback to the insular cortex in mice. Science 374, 1010–1015. 10.1126/science.abj8817 34793231

[B22] Koren T. Yifa R. Amer M. Krot M. Boshnak N. Ben-Shaanan T. L. (2021). Insular cortex neurons encode and retrieve specific immune responses. Cell 184, 5902–5915. e17. 10.1016/j.cell.2021.10.013 34752731

[B23] Lee R. K. Stainier D. Y. Weinstein B. M. Fishman M. C. (1994). Cardiovascular development in the zebrafish. II. Endocardial progenitors are sequestered within the heart field. Development 120, 3361–3366. 10.1242/dev.120.12.3361 7821208

[B24] Libby P. Ridker P. M. Hansson G. K. (2011). Progress and challenges in translating the biology of atherosclerosis. Nature 473, 317–325. 10.1038/nature10146 21593864

[B25] Liu Y. Sun J. (2021). Detection of pathogens and regulation of immunity by the *Caenorhabditis elegans* nervous system. mBio 12, e02301–e02320. 10.1128/mBio.02301-20 33785621 PMC8092265

[B27] Magnon C. Hall S. J. Lin J. Xue X. Gerber L. Freedland S. J. (2013). Autonomic nerve development contributes to prostate cancer progression. Science 341, 1236361. 10.1126/science.1236361 23846904

[B28] Malheiro A. Wieringa P. Moroni L. (2021). Peripheral neurovascular link: An overview of interactions and *in vitro* models. Trends Endocrinol. metabolism TEM 32, 623–638. 10.1016/j.tem.2021.05.004 34127366

[B29] McMahon S. B. La Russa F. Bennett D. L. (2015). Crosstalk between the nociceptive and immune systems in host defence and disease. Nat. Rev. Neurosci. 16, 389–402. 10.1038/nrn3946 26087680

[B30] Mohanta S. K. Yin C. Peng L. Srikakulapu P. Bontha V. Hu D. (2014). Artery tertiary lymphoid organs contribute to innate and adaptive immune responses in advanced mouse atherosclerosis. Circulation Res. 114, 1772–1787. 10.1161/CIRCRESAHA.114.301137 24855201

[B54] Mohanta S. K. Peng L. Li Y. Lu S. Sun T. Carnevale L. (2022). Neuroimmune cardiovascular interfaces control atherosclerosis. Nature 117, 402–410. 10.1038/s41586-022-04673-6 35477759

[B31] Moos M. P. John N. Grabner R. Nossmann S. Gunther B. Vollandt R. (2005). The lamina adventitia is the major site of immune cell accumulation in standard chow-fed apolipoprotein E-deficient mice. Arteriosclerosis, thrombosis, Vasc. Biol. 25, 2386–2391. 10.1161/01.ATV.0000187470.31662.fe 16179593

[B32] Morrison S. F. Nakamura K. (2019). Central mechanisms for thermoregulation. Annu. Rev. Physiol. 81, 285–308. 10.1146/annurev-physiol-020518-114546 30256726

[B33] Olofsson P. S. Rosas-Ballina M. Levine Y. A. Tracey K. J. (2012). Rethinking inflammation: Neural circuits in the regulation of immunity. Immunol. Rev. 248, 188–204. 10.1111/j.1600-065X.2012.01138.x 22725962 PMC4536565

[B34] Singh A. P. Sosa M. X. Fang J. Shanmukhappa S. K. Hubaud A. Fawcett C. H. (2019). αKlotho regulates age-associated vascular calcification and lifespan in zebrafish. Cell Rep. 28, 2767–2776. 10.1016/j.celrep.2019.08.013 31509740

[B35] Srikakulapu P. Hu D. Yin C. Mohanta S. K. Bontha S. V. Peng L. (2016). Artery tertiary lymphoid organs control multilayered territorialized atherosclerosis B-cell responses in aged ApoE-/- mice. Arteriosclerosis, thrombosis, Vasc. Biol. 36, 1174–1185. 10.1161/ATVBAHA.115.306983 PMC4894775 27102965

[B36] Stainier D. Y. Fouquet B. Chen J. N. Warren K. S. Weinstein B. M. Meiler S. E. (1996). Mutations affecting the formation and function of the cardiovascular system in the zebrafish embryo. Development 123, 285–292. 10.1242/dev.123.1.285 9007248

[B37] Steinman L. (2012). Lessons learned at the intersection of immunology and neuroscience. J. Clin. investigation 122, 1146–1148. 10.1172/JCI63493 PMC3314485 22466655

[B38] Sun T. Li Y. Forstera B. Stanic K. Lu S. Steffens S. (2022). Tissue clearing approaches in atherosclerosis. Methods Mol. Biol. 2419, 747–763. 10.1007/978-1-0716-1924-7_45 35237999

[B39] Talbot S. Foster S. L. Woolf C. J. (2016). Neuroimmunity: Physiology and Pathology. Annu. Rev. Immunol. 34, 421–447. 10.1146/annurev-immunol-041015-055340 26907213

[B40] Tam S. J. Watts R. J. (2010). Connecting vascular and nervous system development: Angiogenesis and the blood-brain barrier. Annu. Rev. Neurosci. 33, 379–408. 10.1146/annurev-neuro-060909-152829 20367445

[B41] Tracey K. J. (2012). Immune cells exploit a neural circuit to enter the CNS. Cell 148, 392–394. 10.1016/j.cell.2012.01.025 22304908 PMC4544704

[B42] Udit S. Blake K. Chiu I. M. (2022). Somatosensory and autonomic neuronal regulation of the immune response. Nat. Rev. Neurosci. 23, 157–171. 10.1038/s41583-021-00555-4 34997214 PMC9539447

[B43] Vedder V. L. Aherrahrou Z. Erdmann J. (2020). Dare to compare. Development of atherosclerotic lesions in human, mouse, and zebrafish. Front. Cardiovasc Med. 7, 109. 10.3389/fcvm.2020.00109 32714944 PMC7344238

[B44] Walchli T. Wacker A. Frei K. Regli L. Schwab M. E. Hoerstrup S. P. (2015). Wiring the vascular network with neural cues: A CNS perspective. Neuron 87, 271–296. 10.1016/j.neuron.2015.06.038 26182414

[B45] Wang Z. Zhang X. Lu S. Zhang C. Ma Z. Su R. (2023). Pairing of single-cell RNA analysis and T-cell receptor profiling indicates breakdown of T-cell tolerance checkpoints in atherosclerosis. Nat. Cardiovasc. Res. (In-Press). 10.1038/s44161-023-00218-w PMC10448629 37621765

[B46] Wang Z. Zhang X. Zhang C. Li Y. Lu S. Mohanta S. (2022). Combined single-cell RNA and single-cell α/β T cell receptor sequencing of the arterial wall in atherosclerosis. Methods Mol. Biol. 2419, 727–746. 10.1007/978-1-0716-1924-7_44 35237998

[B47] Weinstein B. M. Stemple D. L. Driever W. Fishman M. C. (1995). Gridlock, a localized heritable vascular patterning defect in the zebrafish. Nat. Med. 1, 1143–1147. 10.1038/nm1195-1143 7584985

[B48] Yin C. Ackermann S. Ma Z. Mohanta S. K. Zhang C. Li Y. (2019). ApoE attenuates unresolvable inflammation by complex formation with activated C1q. Nat. Med. 25, 496–506. 10.1038/s41591-018-0336-8 30692699 PMC6420126

[B49] Yin C. Mohanta S. Maffia P. Habenicht A. (2017). Editorial: Tertiary lymphoid organs (TLOs): Powerhouses of disease immunity. Front. Immunol. 8, 228. 10.3389/fimmu.2017.00228 28321222 PMC5337484

[B50] Zacchigna S. Ruiz de Almodovar C. Carmeliet P. (2008). Similarities between angiogenesis and neural development: What small animal models can tell us. Curr. Top. Dev. Biol. 80, 1–55. 10.1016/S0070-2153(07)80001-9 17950371

[B51] Zhang X. Lei B. Yuan Y. Zhang L. Jin S. (2020). Brain control of humoral immune responses amenable to behavioural modulation. Nature 581, 204–208. 10.1038/s41586-020-2235-7 32405000

[B52] Zhang X. Wang Z. Zhang C. Li Y. Lu S. Steffens S. (2022). Laser capture microdissection-based mRNA expression microarrays and single-cell RNA sequencing in atherosclerosis research. Methods Mol. Biol. 2419, 715–726. 10.1007/978-1-0716-1924-7_43 35237997

[B53] Zych A. D. Gogolla N. (2021). Expressions of emotions across species. Curr. Opin. Neurobiol. 68, 57–66. 10.1016/j.conb.2021.01.003 33548631 PMC8259711

